# Contrasting patterns of genetic and phenotypic divergence of two sympatric congeners, *Phragmites australis* and *P. hirsuta*, in heterogeneous habitats

**DOI:** 10.3389/fpls.2023.1299128

**Published:** 2023-12-12

**Authors:** Tian Qiu, Zhiyuan Liu, Haiyan Li, Ji Yang, Bao Liu, Yunfei Yang

**Affiliations:** ^1^School of Life Sciences, Changchun Normal University, Changchun, China; ^2^Key Laboratory of Vegetation Ecology, Ministry of Education, Institute of Grassland Science, Northeast Normal University, Changchun, China; ^3^College of Computer Science and Technology, Changchun University, Changchun, China; ^4^Ministry of Education Key Laboratory for Biodiversity Science and Ecological Engineering, Fudan University, Shanghai, China; ^5^Key Laboratory of Molecular Epigenetics, Ministry of Education, Northeast Normal University, Changchun, China

**Keywords:** habitat, heterogeneity, *Phragmites hirsuta*, *Phragmites australis*, generalist, specialist, coexistence

## Abstract

Habitat heterogeneity leads to genome-wide differentiation and morphological and ecological differentiation, which will progress along the speciation continuum, eventually leading to speciation. *Phragmites hirsuta* and *Phragmites australis* are sympatric congeners that coexist in saline-alkaline meadow soil (SAS) and sandy soil (SS) habitats of the Songnen Meadow. The results provided genetic evidence for two separate species of reeds. Genetic diversity and spatial genetic structure supported the specialist-generalist variation hypothesis (SGVH) in these two sympatric reed species, suggesting that *P. australis* is a generalist and *P. hirsuta* is a habitat specialist. When we compared these different species with respect to phenotypic and genetic variation patterns in different habitats, we found that the phenotypic differentiation of *P. australis* between the two habitats was higher than that of *P. hirsuta.* Multiple subtle differences in morphology, genetic background, and habitat use collectively contribute to ecological success for similar congeners. This study provided evidence of the two reed congeners, which should contribute to their success in harsh environments.

## 1 Introduction

Habitat heterogeneity leads to genetic and morphological divergence among different populations, eventually leading to speciation (Hume et al., 2018; Cozzolino et al., 2021). Elucidating the mechanisms that give rise to population divergence and eventually initiate speciation is a key step for understanding the evolution of biodiversity. Phenotypic plasticity variation and genetic differentiation are two main ways to adapt to heterogeneous habitats. In addition, niche divergence may enable the coexistence of congeners occupying the same location or habitat, although niche differentiation between coexisting congeners may be subtle or not visually apparent (Porreca et al., 2017). It is important but still not well understood how sympatric congeners can co-occur within the same landscapes and how each species responds to habitat modification.

*Phragmites australis* and *Phragmites hirsuta* are congeneric reeds. ‘White, stiff hairs on leaves and leaf sheaths’ have been described as a characteristic that distinguishes *P. hirsuta* from *P. australis*. *Phragmites hirsuta* Kitag. has always been taxonomically problematic in monographs and papers because whether *P. hirsuta* Kitag. is a variant, or even species, has not been determined (Chinese Academy of Sciences ‘Flora of China’ Editorial Committee, 2004; Fu, 1995). *Phragmites australis* and *P. hirsuta* often coexist in (saline) meadow, wetland, and sand dune habitats and are distributed widely in China (Liu, 2008). Specifically, these pairs coexist in various habitats of the Songnen meadow. Sympatric *P. hirsuta* and *P. australis* pairs make particularly good models for understanding the adaptation of different species (Eller et al., 2020). We have been ecologically studying *P. australis* across the Songnen Meadow since the 1990s, ranging from morphology, phenotypic plasticity, buds, and matter storage of rhizomes to resistance to stresses (Yang and Li, 2003). Height, total biomass, stem and leaf sheath biomass, stem and leaf fraction, rhizome length and biomass, and photosynthesis parameters have been found to differ remarkably among heterogeneous habitats.

The Songnen Meadow in Northeast China is one of the three largest soda saline-alkaline areas in the world, belonging to an agro-pastoral interlocking zone (Yu et al., 2014). Most of the grassland area consists of saline meadow soil and is surrounded by sand dunes. There exists a mosaic of diverse patchy habitats because of the eco-geo-environment of the Songnen Meadow caused by tectonic processes and exogenic causes such as the East Asia monsoon and irrational human activities (Wu et al., 2005). Sandy desertification and land salinization still expanded rapidly in the last 40 years, affecting plant survival and diversification and posing a risk to the ecological environment and economic development.

In this study, we compared growth performance, life history traits, and functional traits as well as genetic diversity between individuals from different habitats and from different species. We aim to understand the growth status and genetic differentiation pattern of these congeneric reeds in saline-alkaline meadow soil (SAS) and sandy soil (SS) habitats and understand whether they respond to environmental change in different ways. This study added to the relatively few investigations addressing the specialist-generalist variation hypothesis (SGVH) on plants.

## 2 Materials and methods

### 2.1 Study area and soil sampling

Samples were collected from the Pasture Ecology Research Station of Northeast Normal University (123°45’E, 44°45’N). The region is in the Songnen Plain, which is characteristic of a temperate, semiarid, and semiwet monsoon climate. The mean annual precipitation is 313–581 mm, most of which falls between June and August (Li et al., 2014). The Songnen Plain in Northeast China belongs to an agro-pastoral interlocking zone and is an alluvial plain that developed on the base of a faulted basin in the Mesozoic. The saline-alkaline land is mainly distributed in the low plains, low terraces, alluvial flats, ancient riverbeds, and oxbow lakes. Although salinized soil is distributed widely in arid and semiarid regions, the Songnen Plain is the only block with such a large area of saline-alkali soil in China. The prime time for sandy desertification was the Late Pleistocene, when localized salt accumulated.

**Figure 1:**
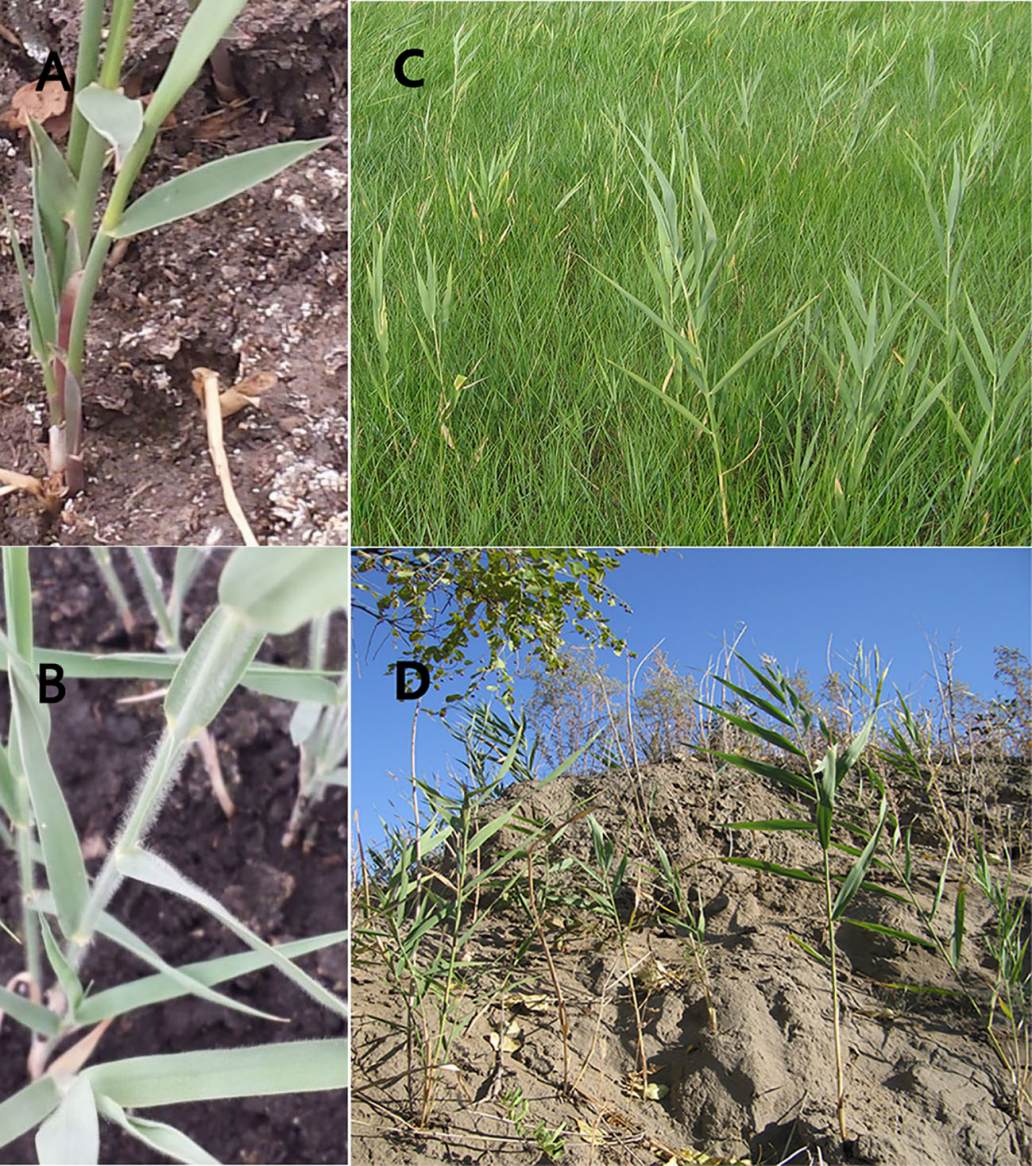
Pictures of *P. australis* **(A)** and *P. hirsuta* **(B)**; two habitats in the study area at the Pasture Ecology Research Station of Northeast Normal University. saline-alkaline meadow soil (SAS) **(C)** and sandy soil (SS) **(D)**.

*Phragmites australis* samples were collected from an approximately 10 × 5-km area within the Pasture Ecology Research Station. The two typical dryland habitats can be described as follows (**Figure 1**). Saline-alkaline meadow soil (SAS) habitat: *Leymus chinensis* and *Phragmites australis* are the typical species in the meadow steppe with a pH of approximately 9.5. There is no accumulated rainwater or extremely short-term accumulation throughout the year. Sandy soil (SS) habitat: *P. australis* is the dominant species in the sandy soil habitat with a pH of 8–8.5 and good soil aeration (Yang and Lang, 1998; Yang and Li, 2003).

Soil samples were collected when individuals were sampled at each site for AFLP (Amplified Fragment Length Polymorphism). They were measured for moisture, pH, electrical conductivity (EC), total phosphorus, total nitrogen, organic matter, NO_3_-nitrogen, NH_4_-nitrogen, Cl^-^, SO_4_^2-^, Na^+^, K^+^, Mg^2+^ and Ca^2+^ (Walky and Black, 1934; Su et al., 2005; Wu et al., 2009; Zhang and Mu, 2009; Li and Wang, 2012).

### 2.2 Plant sampling and phenotyping

**Figure 2:**
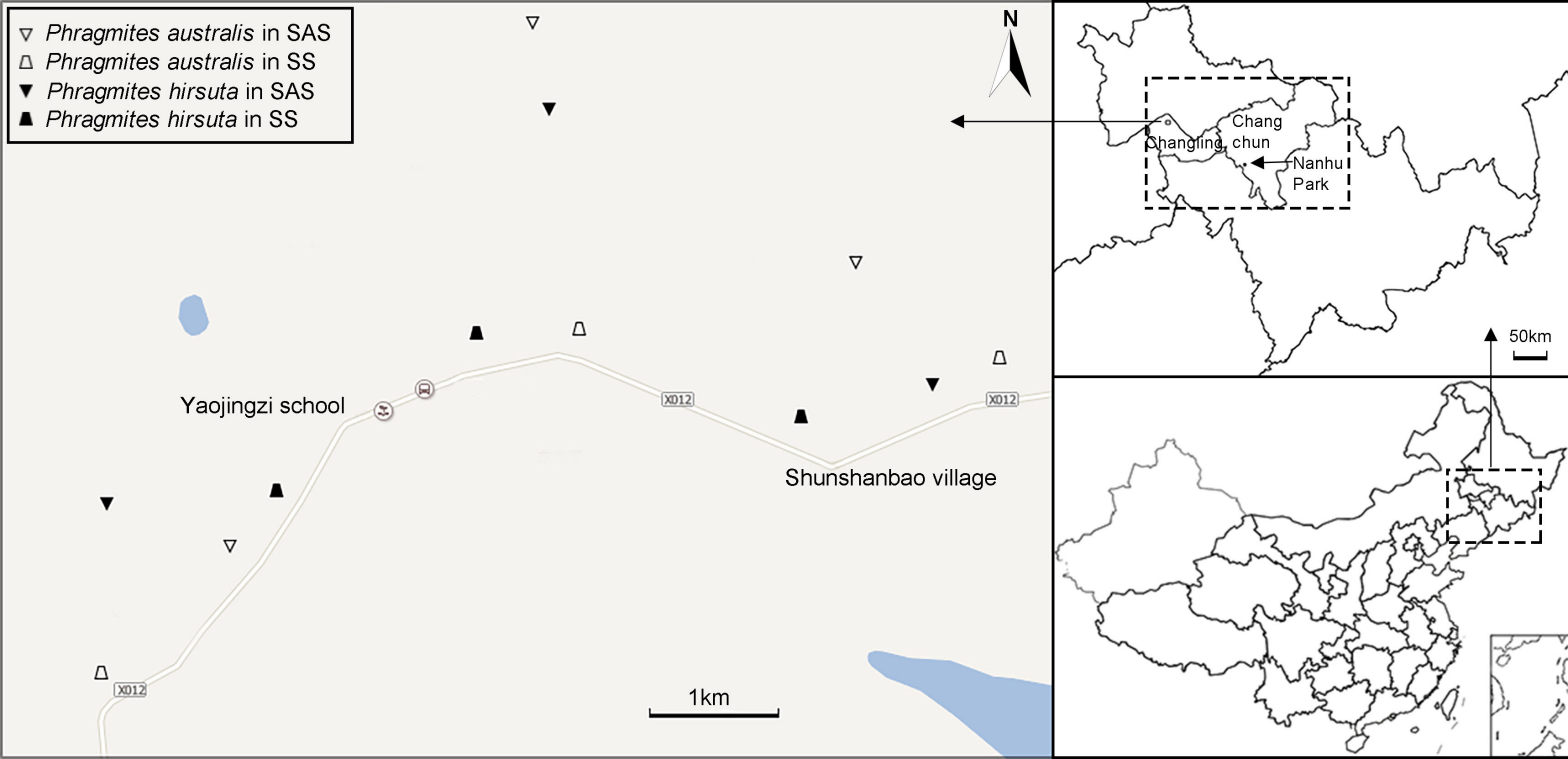
Sampling locations of *Phragmites australis* and *Phragmites hirsuta* in two habitats. Different habitats are indicated by distinct symbols (triangle, saline-alkaline meadow soil (SAS); trapezoid, sandy soil (SS); filled symbols indicate *P. hirsuta*).

Three populations (sites) were sampled (**Figure 2**; **Table S1**). The numbers of individuals at each site were 7, 6, and 7 in SAS habitat and 6, 6, and 8 in SS habitat for *P. australis* and 8, 6, and 7 in SAS habitat and 7, 7, and 6 in SS habitat for *P. hirsuta*. All the individuals sampled were separated by a minimum of 30 m to prevent sampling members of the same clone.

To identify the number of chromosomes, root tips of all samples that were produced from rhizome-grown plants were taken for cytological analysis using an epifluorescence Olympus BX61 microscope.

The aboveground parts were harvested when the individuals had reached maximum biomass by late September, 2022. All the traits of 60 randomly selected individuals from each habitat (20 at each site) were noted. Internode length refers to the length between the 5th and 6th nodes from the top. After the plants were divided into organs, all of them were dried at 80°C to a constant weight, and the dry weight was determined. Three mature, fresh leaves from each one were scanned on an LI-3100 leaf area meter (Li-Cor, Lincoln, NE, USA), oven dried, and weighed to determine specific leaf area [SLA, leaf area (cm^2^)/leaf biomass (g)].

### 2.3 AFLP analysis

Genomic DNA was extracted using a modified cetyltrimethylammonium bromide (CTAB) method (Kidwell and Osborn, 1992). A standard amplified fragment length polymorphism (AFLP) analysis with minor modifications was performed (Vos et al., 1995; Wang et al., 2005). We used fluorescently labeled selective amplification primers at the 5’ end (Applied Biosystems Inc., Foster City, CA, USA), a ROX-500-labeled internal size standard (Applied Biosystems) and an ABI-automated 3730XL DNA capillary sequencer. The 13 EcoRI+3/MseI+3 primer pairs (New England Biolabs, Massachusetts, USA) that provided the most reliable, consistently scorable bands were chosen after screening (**Table S1**). GeneMapper v.4.1 software (Applied Biosystems) was utilized to analyze the raw data. We scored all well-resolved and reliable bands with a binary code: one for band present and zero for band absent. Reliable bands were assessed across duplicates for each sample by consistency of banding patterns. We excluded singleton observations, i.e., markers with only one nonconsensus band, from the dataset. All scoring was performed ‘blindly’ by the same person, who lacked any information about the samples. Nonoverlapping peaks in the 150-500 bp range were included, and differences in intensity were considered. We did not consider bands that appeared in fewer than four individuals. We evaluated genotyping error rates for each primer combination when repeated, and independent analyses of six individuals were performed. Root tips of all 81 sampled individuals of *P. australis* and *P. hirsuta* from the two habitats were analyzed.

### 2.4 Statistical analyses

Associations between soil and plant traits were analyzed using cor.test, cor, corrplot in R. Comparisons between soil or plant characteristics were analyzed with Student’s t test. The relationships between genetic variation and soil for all individuals were calculated via a simple Mantel test.

The levels of genetic diversity were calculated using the POPGENE 1.32 program (Yeh et al., 2000). The parameters included the number of alleles per locus (NA), the effective allele number per locus (NE), the percentage of polymorphic loci (P), Nei’s gene diversity index (HE), the Shannon information index (I) and gene flow (Nm). The percentage of polymorphic bands at the 5% level was calculated by AFLPSURV 1.0 (Vekemans et al., 2002).

An unweighted pair group method with arithmetic mean (UPGMA) analysis was used to build a dendrogram of the relationships among individuals by PAUP4b10 with 1000 bootstrap replicates (Swofford, 2003). Hierarchical AMOVA to identify variance among species, among habitats within species and within habitats or common AMOVA were conducted via GENALEX version 6.5 (Peakall and Smouse, 2006). STRUCTURE 2.3 for the AFLPs was implemented with 10 runs, a burn-in of 10^5^ followed by 10^5^ Markov chain Monte Carlo (MCMC) iterations (Pritchard et al., 2000). BayeScan 2.1 uses differences in allele frequencies between populations to identify candidate loci under selection from dominant binary data via the reversible-jump Monte Carlo Markov chain (MCMC) algorithm (Foll and Gaggiotti, 2008). We used 20 pilot runs, the sample size was set to 5000, and the thinning interval was set to 10. The loci were ranked according to their estimated posterior probability.

To determine whether soil variation influenced the phenotypes, we carried out redundancy analysis (RDA) using the “vegan” library in R (Oksanen, 2015) (https://www.davidzeleny.net/anadat-r/doku.php/en:forward_sel). The significance of the RDA correlations was determined by a Monte Carlo test. The calculation can be simply described as a set of linear regression analyses, where multivariate responses (phenotypes) are regressed against multivariate predictors (environmental variables). To avoid inflation of variance components in RDA, we performed forward variable selection in the R package ADESPATIAL.

## 3 Results

### 3.1 Soil characteristics of distinct habitats

**Figure 3:**
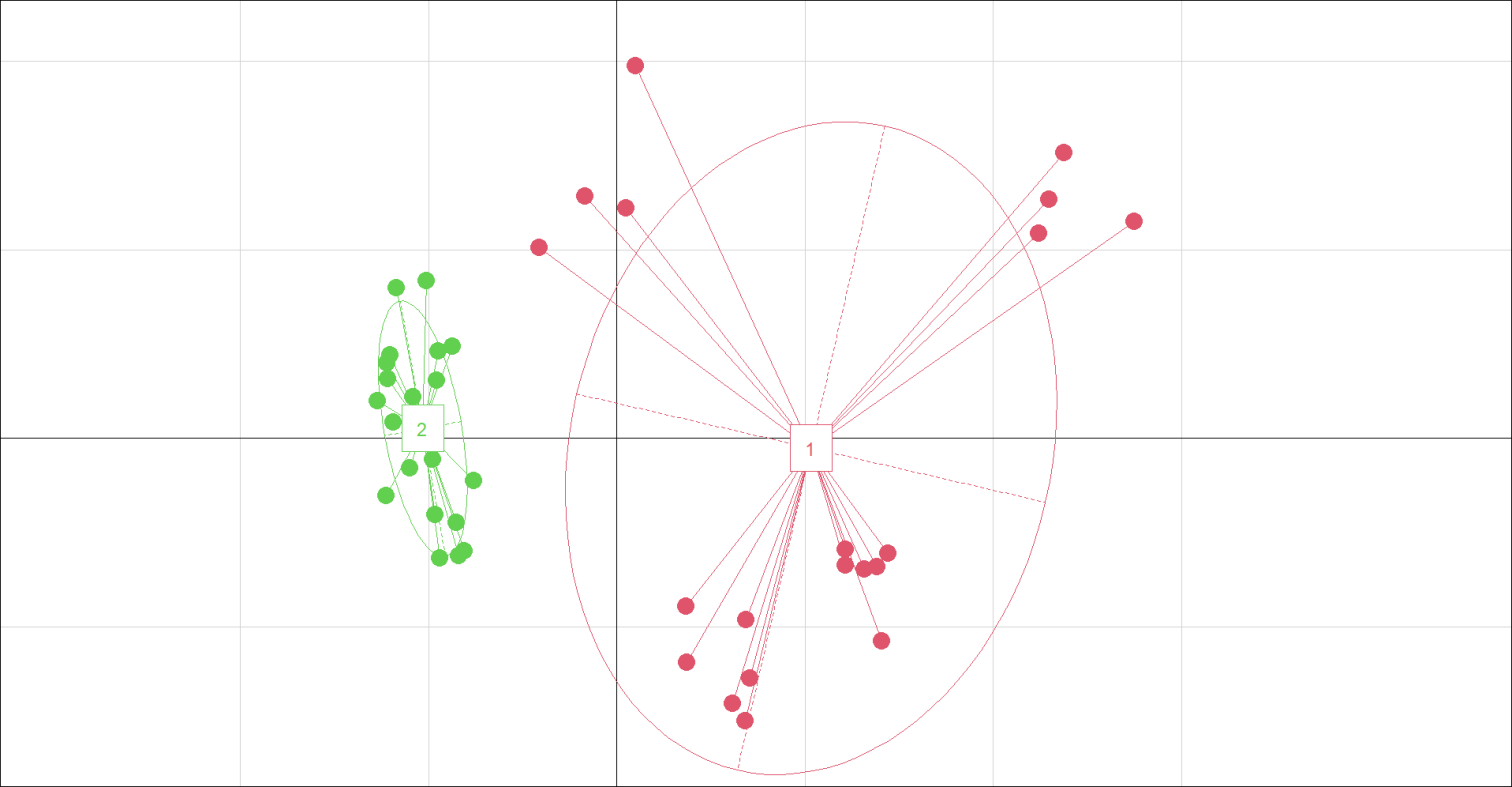
Principal coordinate diagram for soil characteristics of saline-alkaline meadow soil (SAS) (1) and sandy soil (SS) (2). soil characteristics include all 14 parameters. The two axes represent 41.59% (x-axis) and 22.06% (y-axis) of the explained variance.

Various soil traits, including pH, conductivity, Cl^-^, SO_4_^2-^, Na^+^, K^+^, and Ca^2+^, differed significantly between the two habitats by *t* test (**Table S2**). PCA displayed two clear-cut subsets (**Figure 3**). Saline and alkaline stresses (Na^+^, conductivity, K^+^, SO_4_^2-^, pH, Ca^2+^), soil nutrition and water (organic matter, water, total N) are the main factors of soil characteristics according to eigenvectors.

### 3.2 Variation in morphological and growth traits

**Figure 4:**
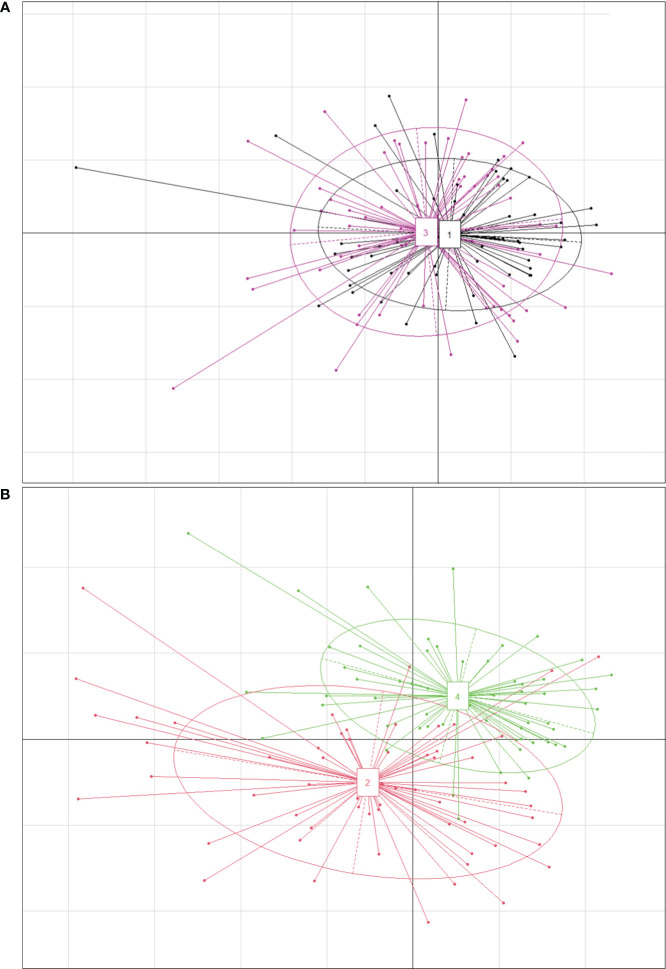
Principal coordinate diagram for phenotypic traits of *P. australis* and *P. hirsuta* individuals in saline-alkaline meadow soil (SAS) **(A)** and sandy soil (SS) **(B)**. All 18 traits were included. “1” in black and “2” in red denote *P. australis.* “3” in pink and “4” in green denote *P. hirsuta*. The two axes represented 35.77% (x-axis) and 16.38% (y-axis) of the explained variance in **(A)** and 46.39% (x-axis) and 16.71% (y-axis) in **(B)**.

There were no significant differences in most traits between *P. australis* and *P. hirsuta* in SAS habitat except maximum leaf width and specific leaf area (SLA), with those of *P. hirsuta* being higher than those of *P. australis*. (**Figure S1**). The values of height, stem diameter, maximum leaf width, node number, leaf number, SLA, leaf, stem, leaf sheath biomass and total biomass from *P. australis* of SS habitat were significantly greater than those from *P. hirsuta.* However, the leaf water content and stem fraction from *P. hirsuta* were significantly greater than those from *P. australis*. Therefore, the differences in most traits between the two reeds in SS were larger than those in SAS, which was consistent with the PCA results (**Figure 4**).

The differences in most traits of *P. australis* between SAS and SS habitats were larger than those of *P. hirsuta* according to the coefficient of variation, means, ANOVA and *t* test except node number and leaf water content (**Table S3**). Two-way ANOVA showed that more traits of *P. australis* were significantly influenced by habitat than those of *P. hirsuta*. More traits of *P. hirsuta* were significantly influenced by population than those of *P. australis* (**Table S4**).

### 3.3 AFLP-based genetic diversity and population structure

**Figure 5:**
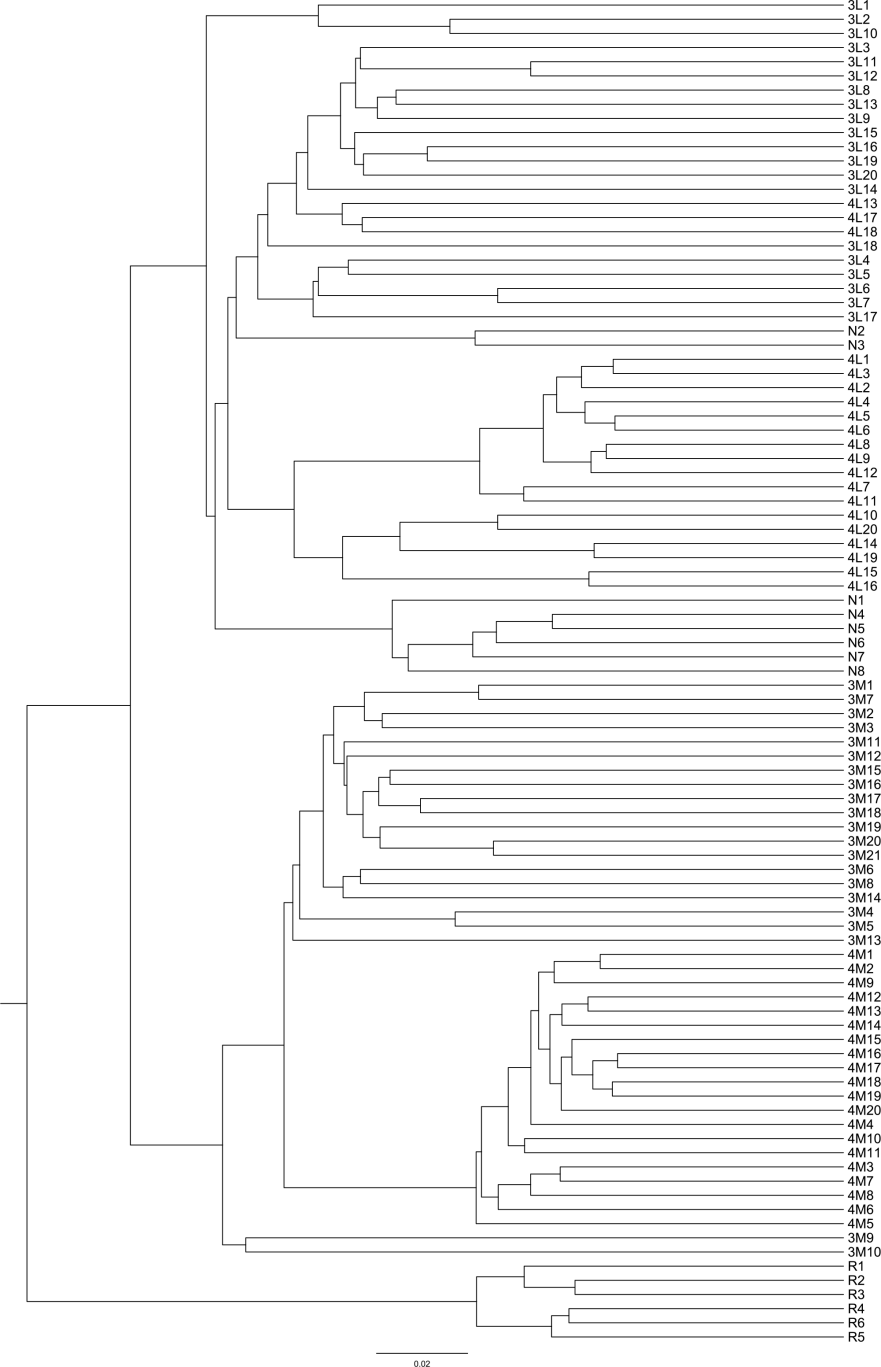
Dendrogram of 95 clones of *Phragmites australis* and *Phragmites hirsuta* in the two habitats at small scales in the Songnen Steppe as well as outgroups constructed by UPGMA. Note: L indicates *Pragmites australis* and M indicates *Phragmites hirsuta*. N indicates *Pragmites australis* in Nanhu Park in Changchun. R indicates *Phragmites japonicus*. The numbers “3” and “4” before “L” or “M” represent habitats of saline-alkaline meadow soil (SAS) and sandy soil (SS), respectively.

**Figure 6:**
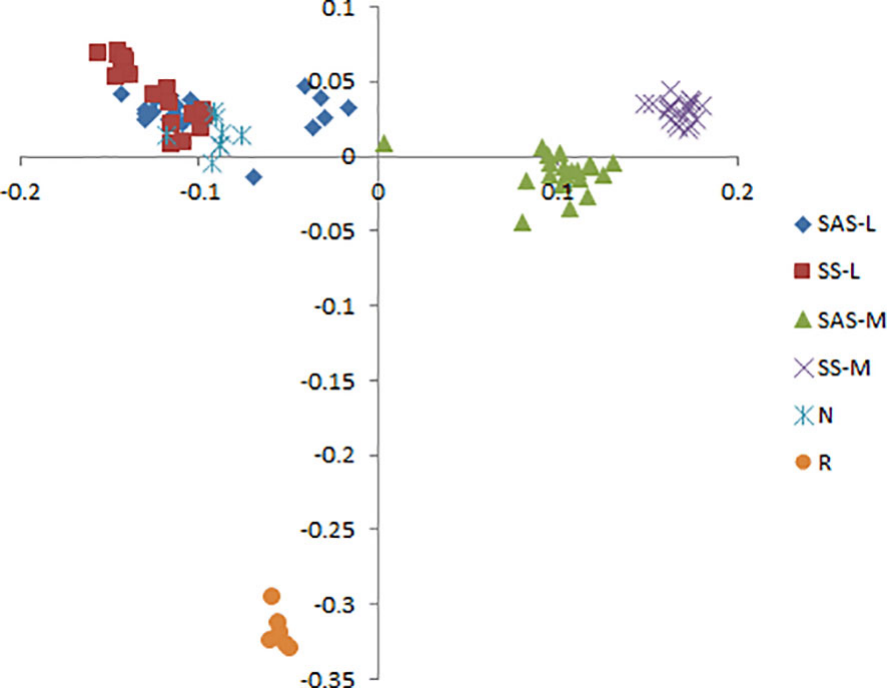
Principal coordinate analysis (PCA) based on AFLP banding patterns showing the relationships among 95 clones of *Phragmites australis*, *Phragmites hirsuta* and outgroups. (L indicates *Pragmites australis* and M indicates *Phragmites hirsuta*. N indicates *Pragmites australis* in Nanhu Park in Changchun. R indicates *Phragmites japonicus*. SAS and SS represent saline-alkaline meadow soil and sandy soil habitat, respectively.

**Figure 7:**
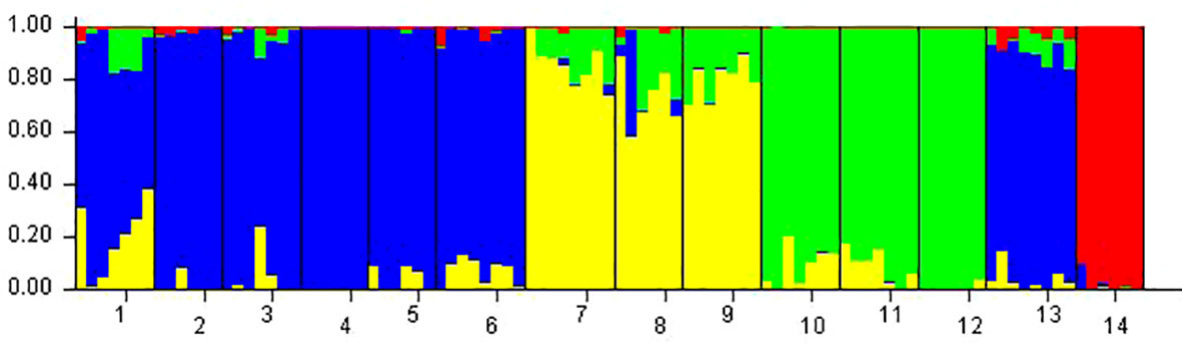
Genetic divergence in the two types of reeds illustrated by STRUCTURE based on AFLPs. Each vertical line represents an individual, and each color represents a cluster in the histograms of STRUCTURE with K=4. Populations were separated by black bars and identified at the bottom of the histogram, with 1, 2, and 3 representing *Phragmites australis* populations in saline-alkaline meadow soil (SAS), 4, 5, and 6 representing populations in sandy soil (SS), 7, 8, and 9 representing *Phragmites hirsuta* populations in SAS, 10, 11, and 12 representing those in SS, 13 representing *P. australis* populations in Nanhu Park of Changchun and 14 representing *Phragmites japonicus*.

*Phragmites australis* showed a higher level of genetic diversity than *P. hirsuta* not only in SAS but also in SS habitat (**Table 1**). The dendrogram indicated that individuals from *P. australis* often grouped together with remote individuals from Changchun city, well separated from *P. hirsuta.* Both reeds were divided into two subsets, i.e., SAS and SS habitats, according to their edaphic origin (**Figure 5**). PCA displayed clear-cut clusters, with the first principal component explaining 18.53% of the variance and the second component explaining 9.27% of the variance (**Figure 6**). The genetic structure analysis showed that individuals of *P. australis*, *P. hirsuta* and the outgroup *Phragmites japonicus* were separated, and the *P. hirsuta* individuals were divided into SAS and SS subsets when K=4 (**Figure 7**).

**Table 1:** Estimates of the genetic diversity of *Phragmites australis* and *Phragmites hirsuta* from two habitats.

Habitat	na	ne	He	I	P
*P. australis* in SAS	1.781 ± 0.414	1.384 ± 0.341	0.235 ± 0.177	0.362 ± 0.246	78.087
*P. australis* in SS	1.705 ± 0.456	1.328 ± 0.336	0.203 ± 0.179	0.315 ± 0.253	70.510
*P. hirsuta* in SAS	1.77 ± 0.419	1.36 ± 0.339	0.221 ± 0.177	0.344 ± 0.246	76.990
*P. hirsuta* in SS	1.514 ± 0.499	1.222 ± 0.311	0.139 ± 0.171	0.219 ± 0.249	51.377

The values of na, ne, He, and I are indicated as the means ± SEs; P, percentage of all loci that are polymorphic regardless of allele frequencies. SAS and SS indicated saline-alkaline meadow soil and sandy soil, respectively.

Hierarchical AMOVA revealed significant genetic differentiation between species, among habitats, and within habitats (all *P* < 0.001) (**Table S5**). AMOVA showed significant genetic differentiation between groups of *P. australis* and *P. hirsuta* (Fst 0.247); the differentiation between SAS and SS habitats of *P. australis* (Fst 0.14) was less than that of *P. hirsuta* (Fst 0.245) (in all cases *P* < 0.001). The candidate loci identified by BayeScan analyses under selection in edaphic differences on Jeffrey’s scale were different (**Table 2**).

**Table 2:** Analysis of molecular variance for groups of *P. australis* and *P. hirsuta* or saline-alkaline meadow soil (SAS) habitat and sandy soil (SS) habitat in each species and BayeScan analyses based on AFLP loci.

Groups	Fst	*P*	Outlier loci
AFLP			
*P. australis* and *P. hirsuta*	0.247	<0.001	
P. australis			
SAS habitat and SS habitat	0.141	<0.001	1990
*P. hirsuta*			
SAS habitat and SS habitat	0.245	<0.001	111

Outlier loci were identified by BayeScan analyses.

We found that except for 8 plants whose chromosomes could not be reliably counted, all the remaining 73 plants had a somatic chromosome number of 2n = 48 (**Figure S2**); hence, aneuploid, ploidy level variation was not the major cause of reproductive isolation and then genetic differentiation between the two species.

### 3.4 Relations between habitat heterogeneity and patterns of genetic and phenotypic divergence

**Figure 8:**
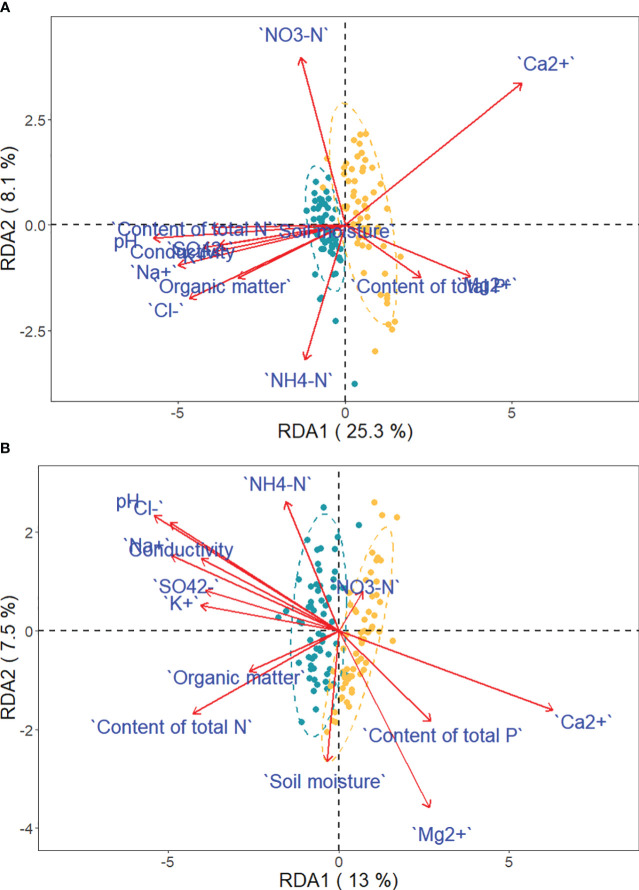
Projection of traits and environmental variables in the RDA, with green and yellow dots representing saline-alkaline meadow soil (SAS) and sandy soil (SS) individuals of *Phragmites australis* **(A)** and *Phragmites hirsuta* **(B)**, respectively.

The RDA model considering the effect of soil on phenotypic traits of *Phragmites australis* among habitats was significant (Monte Carlo test with 999 permutations, F = 6.2648, *P* = 0.001) and accounted for 45.51% of the variance. The biplot showed the distribution of the 120 samples, demonstrating remarkable separation among habitats (**Figure 8**). Likewise, the RDA model of *P. hirsuta* among habitats was significant (F = 4.1236, *P* = 0.001) and accounted for 35.48% of the variance.

Ca^2+^, P, and Mg^2+^ increased along the first axis for both reeds. The factors pH, conductivity, Na^+^, Cl^-^, SO_4_^2-^, and K^+^ were closely correlated. For *P. australis*, pH, conductivity, Na^+^, Cl^-^, SO_4_^2-^, and K^+^ decreased along the second axis, while those traits increased for *P. hirsuta*. The soil Ca^2+^, electrical conductivity (EC), K^+^, organic matter, NH_4_-nitrogen and pH were significant or marginally significant for *P. australis* (**Table 3**), while (marginally) significant factors were the content of total N, soil moisture and organic matter for *P. hirsuta* (α=0.1) (**Table 4**). NH_4_-nitrogen and NO_3_-nitrogen had more positive effects on *P. hirsuta* than on *P. australis.*

**Table 3:** List of measured soil factors and their contribution to traits as determined by forward variable selection with RDA of *P. australis*.

variables	R^2^	F	P value
Ca^2+^	0.243521	37.985797	**0.001**
Organic matter	0.017794	2.818411	**0.08**
NH_4_-N	0.018293	2.945564	**0.085**
pH	0.018163	2.974373	**0.087**
Cl^-^	0.007437	1.220275	0.267
NO_3_-N	0.010642	1.757731	0.177
Mg^2+^	0.006103	1.00806	0.3
Content of total P	0.008346	1.38336	0.24
Soil moisture	0.010682	1.78306	0.18
Na^+^	0.012224	2.060112	0.143
Conductivity	0.046524	8.370469	**0.002**
K^+^	0.032529	6.13051	**0.009**
SO_4_^2-^	0.007192	1.360028	0.213
Content of total N	0.008386	1.594629	0.2

Significance in forward selection was determined by permutation tests. P values less than 0.1 are bolded.

**Table 4:** List of measured soil factors and their contribution to traits as determined by forward variable selection with RDA of *Phragmites hirsute*.

variables	R^2^	F	P value
Content of total N	0.024528	2.9671347	**0.065**
Soil moisture	0.027069	3.3393682	**0.084**
Content of total P	0.006912	0.8515734	0.376
pH	0.014506	1.799591	0.188
Organic matter	0.022455	2.8300412	**0.09**
Mg^2+^	0.008535	1.0764146	0.312
SO_4_^2-^	0.007098	0.8943823	0.284
K^+^	0.01581	2.009945	0.166
NH_4_-N	0.009244	1.1770984	0.302
NO_3_-N	0.010392	1.3271837	0.262
Na^+^	0.002448	0.3107359	0.636
Ca^2+^	0.006473	0.8200998	0.345
Cl^-^	0.005955	0.7527966	0.424
Conductivity	0.00104	0.1303939	0.701

P values less than 0.1 are bolded.

Correlations between separate phenotypic traits and soil characteristics for all individuals were remarkably different between *P. australis* and *P. hirsuta* (**Figure S3**). The relationships between genetic variation and soil factors were also different between the two reeds (**Table S6**).

## 4 Discussion

### 4.1 Genetic evidence for two separate species of reeds

*Phragmites hirsuta* Kitag. has always been taxonomically problematic in monographs and papers. There was no research published on the species delimitation of *P. hirsuta*. According to the Flora of China, there are now 3 species in the genus *Phragmites*: *Phragmites australis* (Cav.) Trin. ex Steud., *Phragmites japonica* Steud. and *Phragmites karka* (Retz.) Trin. ex Steud. Paradoxically, according to *Key to the plants from Northeast China* and *Herbaceous flora of Northeast China*, there are 3 species in the genus *Phragmites* in Northeast China: *Phragmites australis* (Cav.) Trin., *Phragmites japonica* Steud. and *Phragmites hirsuta* Kitag. Whether *P. hirsuta* Kitag. is a variant of *P. australis* or a separate species remained unclear. Since saline meadow soil and sandy soil habitats are putatively contrasting habitats, it has been frequently postulated that they may represent a hot spot of sympatric species suffering divergent selection, such as chalk and basalt soil populations (Li et al., 2020). If our data are somewhat consistent with this hypothesis, *P. australis* and *P. hirsuta* of saline-alkaline meadow soil (SAS) would cluster in one clade, separated from those of the sandy soil (SS), in which *P. hirsuta* would be recognized as a variant. However, the results consistently showed that individuals of *P. hirsuta* and *P. australis* were clear-cut clusters, indicating that *P. hirsuta* can be recognized as being at the species level overriding edaphic selection.

A leaf hair (trichome) often protects individuals against herbivores, desiccation, pathogenic microorganisms, UV light, freezing, etc. Trichome variations are generally adaptive and result from natural selection (Nogueira et al., 2013). Studies such as those in Bignoniaceae, Asclepias L., and Brassicaceae have allowed the use of trichome evolution patterns in phylogenetic analysis (Nogueira et al., 2013; Fishbein et al., 2018; Zhang et al., 2018). The persistence of phenotypic divergence even in sympatric spots, even if we cannot fully exclude some genetic admixture, suggests that some isolating barriers should exist between the two species. Simple trait architectures or polygenic architectures that affect ecological performance and/or mate choice can promote rapid and stable speciation in sympatry with gene flow, as previously suggested (Kautt et al., 2020).

### 4.2 Distinct responses to habitat heterogeneity between species

At the interhabitat level, the genetic differentiation of *P. australis* was lower than that of *P. hirsuta*, whereas the phenotypic differentiation of *P. australis* was higher than that of *P. hirsuta.* Therefore, it is suggested that *P. australis* is a generalist and *P. hirsuta* is a habitat specialist, which is consistent with the specialist-generalist variation hypothesis (SGVH) (Pasinelli, 2022). At the intrahabitat level, *P. australis* showed a higher level of genetic diversity than *P. hirsuta.* Two sympatric woodpecker and two Caenorhabditis species showed that specialist species possessed lower genetic diversity than related generalists (Li et al., 2014; Pasinelli, 2022). Therefore, genetic diversity and spatial genetic structure supported the specialist-generalist variation hypothesis (SGVH). This is because populations of habitat specialists have lower genetic diversity and are genetically more differentiated due to reduced gene flow compared to populations of generalists, as posited by SGVH (Li et al., 2014). The fact that more traits of *P. hirsuta* were influenced by population significantly than *P. australis*, while more traits of *P. australis* were influenced by habitat, also suggested the specialization of *P. hirsuta*.

The SGVH has received some attention in invertebrate, terrestrial vertebrate, and fish species and across specialization types, including habitat, diet, host, and dispersal specificities (Lu et al., 2011; Li et al., 2014; Hoyo and Tsuyuzaki, 2015; Porreca et al., 2017; Titus and Daly, 2017; Wort et al., 2019; Zhao and Duffy, 2019; Pasinelli, 2022). However, few studies have focused on plants. Both specialists and generalists have advantages and disadvantages. Generalists typically use a broad range of resources, while specialists are restricted to a narrow set of resources. The generalist has more habitat available; however, it faces a local competitive disadvantage when meeting a specialist in a patch (Pasinelli, 2022). Thus, for most traits, there were no significant differences between the two reeds in SAS; most growth, biomass traits and SLA from *P. australis* of SS were significantly greater than those from *P. hirsuta*. For specialists, in addition to high competitive ability, stress-tolerant species may occur in a narrower range of environments and be more specialized (Denelle et al., 2020). Indeed, the maximum leaf width and SLA of *P. hirsuta* were higher than those of *P. australis* in SAS; the stem fraction and leaf water content were higher in SS. However, stochastic extinctions of local populations are more likely to occur due to the smaller population sizes. *Phragmites australis* may also make full use of resources distributed in a wide range of habitats, such as by increasing SLA, thereby displaying more distinct phenotypes across two habitats than *P. hirsuta*. Phenotypic variation may not have a heritable basis, as observed in four species of Brazilian frogs (Li et al., 2014). This may explain the discordance between genetic and morphological differentiation in this paper. The species boundary was clear morphologically between *Phrynocephalus guinanensis* and *P. vlangalii*, but the boundary was not so clear genetically, consistent with our result (Hu et al., 2019).

According to the niche-width variation hypothesis that is related to SGVH (Valen, 1965), populations growing in a broader niche are hypothesized to exhibit more variability than populations inhabiting a narrower niche. This could possibly explain why genetic diversity in SAS was higher than that in SS for both reeds, and the diversity of *P. australis* was higher than the corresponding value of *P. hirsuta*. This is because the areas of SAS distributed are larger than those of SS given the fact that the sandy desertification area is 1.12×10^6^ ha while the salinization area is 2.43×10^6^ ha, and because *P. australis* is a generalist (Zhang and Wang, 2001). The availability of multiple resources will reduce interspecific competition and make species with broader niches have higher morphological variation. Accordingly, the niche-width variation hypothesis offers an adaptive explanation for differences in phenotypic variation among SAS and SS between the two reeds.

### 4.3 Coexistence of sympatric congeners

The coexistence of sympatric congeners often stems from a single difference or limiting similarity, allowing niche separation among these closely related competitors (Porreca et al., 2017). Although niche differentiation may be subtle or not visually apparent, we could find some signatures (Porreca et al., 2017). Differences between congeners may be intimately related to the ecological environments reflected in multiple physiological metabolisms, resource acquisition strategies across time or space, adaptability, epigenetic variation and so on. Phenotypic plasticity variation and genetic differentiation play an important role in facilitating those differences, and in turn adaptive evolution and ecological success among sympatric congeners, manifesting in many physiological, morphological, or life-history traits. The phenotypic differentiation of *P. australis* between the two habitats was higher than that of *P. hirsuta*, although the genetic differentiation was lower. This extra phenotypic variation could thus be attributable to phenotypic plasticity. The RDA results indicated that the correlations between morphology and soil factors were different between *P. australis* and *P. hirsuta*. It was suggested that *P. hirsuta* was tolerant of saline-alkaline conditions and that these two reeds have different resource acquisition strategies. Some papers published have explored the genetic basis of those phenotypic differences. It was reported that approximately 7% of the proteome explained the genetic factors rather than phenotypic plasticity and thereby contributed to differences between two sympatric marine snail ecotypes (Martínez-Fernández et al., 2010). Among the five Gulf Coast beach mouse subspecies, the two most genetically divergent subspecies occupy the most similar habitats. The changes in *Mc1r* allele frequency contribute to their fine-tuned pigment patterns to match the local environment (Mullen et al., 2009). Among two congeneric rocky intertidal gastropods, a habitat generalist showed a more continuous distribution, higher abundances and lower genetic differentiation compared to the specialist (Wort et al., 2019). As for epigenetic basis, seven pairs of congeneric plants from the Cazorla Mountains showed partial consistency with the expectation that higher epigenetic diversity could alleviate the lower genetic diversity in endemic plants (Medrano et al., 2020). Additionally, other papers have reported the differences in physiological, morphological, or life-history traits between sympatric congeners. Differences in the metabolic rate and internal and external morphology of endangered pallid sturgeon and a more abundant sympatric congener, the shovelnose sturgeon, led to their success (Porreca et al., 2017). The cichlid fishes of barred populations differ in body shape from their nonbarred sympatric congeners. Color is genetically correlated with ecology (Kusche et al., 2015; Kautt et al., 2020). No overlap in diet underlying the coexistence of two rosefinch congeners has facilitated their success in extreme environments (Lu et al., 2011). Specifically, some sympatric congeners show differences in reproduction. Experiments found that the two palms have different flowering times and soil preferences and strong temporal segregation in the patrolling activity of males between two sympatric Morpho species (Le Roy et al., 2021). The pollination system specialization enables the co-occurrence of closely related *Achimenes* species (Ramírez-Aguirre et al., 2019). *Drosera anglica* and its sympatric congener *D. rotundifolia* populations are primarily maintained by vegetative and sexual reproduction, respectively (Hoyo and Tsuyuzaki, 2015). Two orchid ecotypes with different phenotypic floral divergence showed different habitat preferences (Cozzolino et al., 2021).

### 4.4 Metapopulation model of multiple-habitat landscape

The coexistence of habitat specialist and generalist species is widely observed at the landscape scale. However, in a coarse-grained heterogeneous environment, all species have a metapopulation structure in a landscape consisting of patches of different habitat types. Generalists are often described as opportunistic species, while specialists are recognized as highly competitive. Species are often faced with a trade-off between performing a few activities well (specialists) and performing many activities poorly (generalists) or a competition/colonization trade-off. Specialists are currently declining worldwide compared with generalists due to habitat loss and fragmentation, which seriously influence species diversity, whereas generalists can ensure persistence. Generalists could also be favored when specialists are maintained below their habitat carrying capacity, such as by environmental disturbance. However, when the environment contains many patches, the high propagule pressure of the specialists can exclude the generalists. The balance occurs from the turnover in the production of specialists and generalists, speciation, and extinction. Habitat selection in relative habitat quality is not necessary for coexistence (Nagelkerke and Menken, 2013). Increased niche width, dispersal ability or local competitive ability of the generalist enhances its performance compared to the specialists. Both species’ life-history traits and environmental factors (disturbance, spatial heterogeneity, and autocorrelation) are expected to influence the coexistence of specialist and generalist species (Büchi and Vuilleumier, 2014).

Using an integrative approach, we unraveled empirical evidence of the comparison and relations of genetic materials, phenotypes, and environment between two sympatric reed congeners and suggested the existence of a selection pressure responsible for phenotypic divergence. The reproductive isolation of *P. australis* and *P. hirsuta* is, as yet, insufficiently understood to determine the mechanism underlying coexistence. As high-throughput methods in molecular ecology with well-studied reproductive ecology continue to grow, our study provides a key underpinning for exploring the precise evolutionary mechanisms driving sympatric existence and evolution.

## Data availability statement

The datasets presented in this study can be found in online repositories. The names of the repository/repositories and accession number(s) can be found below: https://doi.org/10.5061/dryad.8sf7m0cw2, https://datadryad.org/stash/share/wpq7lZISSBR_OLiUn4wsoHJwI7kvwKPaeI-O-WqOLjs.

## Author contributions

TQ: Conceptualization, Methodology, Project administration, Writing – original draft. ZL: Conceptualization, Methodology, Writing – review & editing. HL: Writing – review & editing. JY: Conceptualization, Methodology, Writing – review & editing. BL: Conceptualization, Methodology, Writing – review & editing. YY: Conceptualization, Methodology, Writing – review & editing.
