# Supplementary material for: Contrasting patterns of genetic and phenotypic divergence of two sympatric congeners, Phragmites australis and P. hirsuta, in heterogeneous habitats
Source: Front Plant Sci. 2023 Dec 12;14:1299128. doi: 10.3389/fpls.2023.1299128 (PMC10756910; doi:10.3389/fpls.2023.1299128)
Supplement: Supplementary file 1 [file DataSheet_1.docx]

***Supplementary Material***

**Table S****1** The adaptors, preselective primers, and primer pairs for selective amplification used for the AFLP analyses.

|  | | | | Sequence | | | | | | | | | |  |  |
| --- | --- | --- | --- | --- | --- | --- | --- | --- | --- | --- | --- | --- | --- | --- | --- |
| Adaptor | | | |  | | | | | | | | | |  |  |
| *EcoR*Ⅰ-adaptor 1 | | | | 5'-CTCGTAGACTGCGTACC-3' | | | | | | | | | |  |  |
| *EcoR*Ⅰ-adaptor 2 | | | | 5'-AATTGGTACGCAGTCTAC-3' | | | | | | | | | |  |  |
| *Mse*Ⅰ-adaptor 1 | | | | 5'-GACGATGAGTCCTGAG-3' | | | | | | | | | |  |  |
| *Mse*Ⅰ-adaptor 2 | | | | 5'-TACTCAGGACTCAT-3' | | | | | | | | | |  |  |
| Pre-selective primer | | | | | | | | | | | | | |  |  |
| *EcoR*Ⅰ+A | | | | 5'-GACTGCGTACCAATTCA-3' | | | | | | | | | |  |  |
| *Mse*Ⅰ+C | | | | 5'-GATGAGTCCTGAGTAAC-3' | | | | | | | | | |  |  |
| *EcoR*Ⅰ+3 primer | | | | | | | | | | | | | |  |  |
| a. *E*-AAC | | | | 5'-GACTGCGTACCAATTCAAC-3' | | | | | | | | | |  |  |
| b*. E-*AAG | | | | 5'-GACTGCGTACCAATTCAAG-3' | | | | | | | | | |  |  |
| c. *E*-ACA | | | | 5'-GACTGCGTACCAATTCACA-3' | | | | | | | | | |  |  |
| i. *E*-AGA | | | | 5'-GACTGCGTACCAATTCAGA-3' | | | | | | | | | |  |  |
| j. *E*-ATC | | | | 5'-GACTGCGTACCAATTCATC-3' | | | | | | | | | |  |  |
| *Mse*Ⅰ+3 primer | | | | | | | | | | | | | |  |  |
| 2. *M*-CAC | | | | 5'-GATGAGTCCTGAGTAACAC-3' | | | | | | | | | |  |  |
| 3. *M*-CAG | | | | 5'-GATGAGTCCTGAGTAACAG-3' | | | | | | | | | |  |  |
| 4. *M*-CAT | | | | 5'-GATGAGTCCTGAGTAACAT-3' | | | | | | | | | |  |  |
| 5. *M*-CTA | | | | 5'-GATGAGTCCTGAGTAACTA-3' | | | | | | | | | |  |  |
| 6. *M*-CTC | | | | 5'-GATGAGTCCTGAGTAACTC-3' | | | | | | | | | |  |  |
| 7. *M*-CTG | | | | 5'-GATGAGTCCTGAGTAACTG-3' | | | | | | | | | |  |  |
| 8. *M*-CTT | | | | 5'-GATGAGTCCTGAGTAACTT-3' | | | | | | | | | |  |  |
| primer combinations | | | | | | | | | | | | | |  |  |
| *EcoR*Ⅰ+3 | a | b | c | | i | c | i | i | a | j | j | j | j | | i |
| *Mse*Ⅰ+3 | 7 | 4 | 3 | | 4 | 4 | 5 | 2 | 5 | 6 | 2 | 7 | 8 | | 7 |

**Table S2** Soil characteristics in two different habitats of the field survey and the comparison (M±SE) Note: Asterisks indicate the significance of pairwise comparisons (Student’s *t* test; *P<0.05).

| Soil characteristics | saline-alkaline meadow soil (SAS) | sandy soil (SS) |  |
| --- | --- | --- | --- |
| Soil moisture (%) | 6.34±0.76 | 6.53±0.27 |  |
| pH | 9.54±0.16 | 8.47±0.06 | * |
| Conductivity (us cm^-1^) | 189.38±34.69 | 75.94±2.52 | * |
| Content of total P (g kg^-1^) | 0.13±0.01 | 0.17±0.01 |  |
| Content of total N (g kg^-1^) | 0.7±0.08 | 0.6±0.04 |  |
| Organic matter (g kg^-1^) | 6.68±1.11 | 5.29±0.47 |  |
| NO_3_-N (mg kg^-1^) | 5.78±0.49 | 4.62±0.4 |  |
| NH_4_-N (mg kg^-1^) | 7.21±0.83 | 9.13±0.92 |  |
| Cl^-^ (mg L^-1^) | 5.6±0.27 | 3.09±0.23 | * |
| SO_4_^2-^ (mg L^-1^) | 3.73±0.62 | 1.74±0.14 | * |
| Na^+^ (mg L^-1^) | 25.96±4.15 | 2.4±0.41 | * |
| K^+^ (mg L^-1^) | 10±1.86 | 4.36±0.28 | * |
| Mg^2+^ (mg L^-1^) | 1.31±0.41 | 2.44±0.27 |  |
| Ca^2+^ (mg L^-1^) | 0.7±0.08 | 2.73±0.21 | * |

**Table S3** Comparison between *P. australis* and *P. hirsuta* with respect to interhabitats

|  | Coeffecient of variation (individual level, n=120) | | Coeffecient of variation (population level, n=6） | | Difference between means of two habitats | | Percentage of variability ^a^ | | Student’s *t* test ^b^ | |
| --- | --- | --- | --- | --- | --- | --- | --- | --- | --- | --- |
|  | *P. australis* | *P.*  *hirsuta* | *P. australis* | *P. hirsuta* | *P. australis* | *P. hirsuta* | *P. australis* | *P. hirsuta* | *P. australis* | *P. hirsuta* |
| Height (cm) | 0.1816 | 0.1417 | 0.1335 | 0.0818 | 16.5 | 7.9 | 0.2564 | 0.1014 | * | * |
| diameter(mm) | 0.1832 | 0.1537 | 0.0968 | 0.0743 | 0.3393 | 0.1173 | 0.1406 | 0.0272 | * | ns |
| Max-leaf length (cm) | 0.1954 | 0.1886 | 0.1544 | 0.1354 | 6.89 | 4.323 | 0.4543 | 0.1930 | * | * |
| Max-leaf width (cm) | 0.3499 | 0.1833 | 0.3410 | 0.1174 | 0.927 | 0.197 | 0.7623 | 0.1847 | * | * |
| Node number | 0.1252 | 0.1461 ^c^ | 0.0760 | 0.0980 | 1.2 | 1.93 | 0.1569 | 0.3102 | * | * |
| Internode length (cm) | 0.2299 | 0.2878 | 0.1475 | 0.1766 | 1.394 | 1.543 | 0.3164 | 0.2229 | * | * |
| Leaf number | 0.1722 | 0.1499 | 0.095 | 0.0749 | 1.17 | 0.19 | 0.1316 | 0.0046 | * | ns |
| Leaf biomass (g) | 0.4679 | 0.3764 | 0.4098 | 0.2634 | 0.9389 | 0.4097 | 0.5825 | 0.2630 | * | * |
| Stem biomass (g) | 0.4998 | 0.3631 | 0.3218 | 0.1959 | 0.3304 | 0.0824 | 0.1419 | 0.0225 | * | ns |
| Leaf sheath biomass (g) | 0.4174 | 0.3981 | 0.2891 | 0.2104 | 0.2115 | 0.0531 | 0.2048 | 0.0191 | * | ns |
| Inflorescence biomass (g) | 1.7679 | 1.6977 | 0.7215 | 0.7244 | 0.0004 | 0.0338 | 0 | 0.0223 | ns | ns |
| Total biomass (g) | 0.4442 | 0.3353 | 0.3427 | 0.1961 | 1.4812 | 0.4056 | 0.3507 | 0.0654 | * | * |
| Stem fraction | 0.1935 | 0.1641 | 0.1200 | 0.0762 | 0.057 | 0.0299 | 0.2240 | 0.0813 | * | * |
| Leaf sheath fraction | 0.1616 | 0.2528 | 0.1128 | 0.1735 | 0.0343 | 0.0540 | 0.2713 | 0.275 | * | * |
| Inflorescence fraction | 1.6213 | 1.5609 | 0.5851 | 0.7218 | 0.0095 | 0.0158 | 0.025 | 0.0452 | ns | * |
| Leaf fraction | 0.1715 | 0.1963 | 0.1365 | 0.1407 | 0.1008 | 0.0997 | 0.4024 | 0.3211 | * | * |
| Leaf water content | 0.4077 | 0.5172 | 0.1997 | 0.4742 | 0.0726 | 0.2696 | 0.1470 | 0.6860 | * | * |
| SLA | 0.2694 | 0.5671 | 0.2010 | 0.1753 | 59.0678 | 31.4418 | 0.2569 | 0.0154 | * | ns |

^a^ Values are based on SS_habitat_/SS_total_, where SS_habitat_ is the sum of squares between saline-alkaline meadow soil (SAS) and sandy soil (SS) and SStotal is the total variability identified from one-way ANOVA tests. ^b^ * P<0.05, ns, nonsignificant.

**Table S4** F-values from Two-way ANOVA of *P. australis* and *P. hirsuta*

|  |  | *P. australis* | |  |  | | *P. hirsuta* | |  | |
| --- | --- | --- | --- | --- | --- | --- | --- | --- | --- | --- |
|  | Habitat | Population | Habitat×Population | Habitat | | Population | | Habitat×Population | |  |
| Height | **53.574***** | 0.586 ns | **20.083***** |  | **16.047***** | | **8.698***** | | **5.433**** | |
| Diameter | **20.942***** | 0.46 ns | **6.526**** |  | 3.863 ns | | **3.319*** | | **8.674***** | |
| Max. leaf length | **109.003***** | 1.21 ns | **7.269**** |  | **38.806***** | | **16.034***** | | **8.093**** | |
| Max. leaf width | **430.867***** | **3.953*** | **6.227**** |  | **32.145***** | | **8.258***** | | **5.64**** | |
| Node number | **25.934***** | 2.566 ns | **10.1***** |  | **56.89***** | | 0.385 ns | | **5.864**** | |
| Internode length | **55.152***** | 2.453 ns | 0.133 ns |  | **37.182***** | | **6.504**** | | 1.295 ns | |
| Leaf number | **20.152***** | **4.952**** | **4.557*** |  | 0.66 ns | | **13.567***** | | 1.216 ns | |
| Leaf biomass | **186.842***** | 1.158 ns | **8.792***** |  | **50.959***** | | 1.486 ns | | **12.894***** | |
| Stem biomass | **24.811***** | 2.405 ns | **15.645***** |  | 3.382 ns | | **8.675***** | | **8.1**** | |
| Leaf sheath biomass | **39.12***** | **6.097**** | **12.836***** |  | 2.869 ns | | **10.145***** | | **5.917**** | |
| Inflorescence biomass | 0 ns | **4.338*** | **4.937**** |  | 3.032 ns | | 1.341 ns | | **7.44**** | |
| Total biomass | **79.985***** | 2.975 ns | **14.078***** |  | **10.453**** | | **4.889**** | | **12.863***** | |
| Stem fraction | **37.94***** | 0.563 ns | **7.695**** |  | **11.274**** | | **5.669**** | | 1.324 ns | |
| Leaf sheath  fraction | **52.977***** | **7.163**** | **5.832**** |  | **51.594***** | | **8.701***** | | 2.881 ns | |
| Inflorescence fraction | 2.916 ns | 2.964 ns | 2.584 ns |  | **6.708*** | | 0.743 ns | | **8.392***** | |
| Leaf fraction | **98.245***** | **4.132*** | **11.73***** |  | **64.534***** | | **10.685***** | | 0.34 ns | |
| Leaf water  content | **20.973***** | 1.333 ns | 2.585 ns |  | **266.437***** | | 2.68 ns | | 1.271 ns | |
| SLA | **55.004***** | **14.352***** | **8.21***** |  | 1.909 ns | | 0.89 ns | | **3.132*** | |

**Table S5** Results of hierarchical AMOVA with respect to *Phragmites australis* and *Phragmites hirsuta* within and among habitats based on AFLP loci.

| Source | d.f. | | SS | Variance components | % variation | *P* value |
| --- | --- | --- | --- | --- | --- | --- |
| Among species | | 1 | 4394.816 | 69.148 | 17 | *P*<0.001 |
| Among habitats within species | | 2 | 3188.334 | 65.185 | 16 | *P*<0.001 |
| Within habitats | | 77 | 21140.974 | 274.558 | 67 | *P*<0.001 |
| Total | | 80 | 28724.123 | 408.891 | 100 |  |
| PhiPT=0.329 | | |  |  |  |  |

**Table S6.** The relationships between genetic variation and habitat for all individuals across all sites in habitats saline-alkaline meadow soil (SAS) and sandy soil (SS) as well as in both habitats were calculated via a simple Mantel test. The *P* value and r (correlation coefficient) are shown. The *P* values for the relationships between genetic variation and all soil parameters in the combined habitats were all 0.0001 for two reeds, thus they were not showed. The r values of *P. hirsuta* were all larger than those of *P. australis* in the combined habitats.

| Variable | saline-alkaline meadow soil (SAS) | | | | sandy soil (SS) | | | | Both habitats | |
| --- | --- | --- | --- | --- | --- | --- | --- | --- | --- | --- |
|  | *P. australis* n=20 |  | *P. hirsuta* n=21 |  | *P. australis* n=20 |  | *P. hirsuta*  n=20 |  | *P. australis* n=40 | *P. hirsuta*  n=41 |
|  | *r* | *P* | *r* | *P* | *r* | *P* | *r* | *P* | *r* | *r* |
| Soil moisture | 0.2832 | **0.0002** | 0.0230 | 0.3635 | -0.0471 | 0.3705 | 0.3078 | **0.0022** | 0.2812 | 0.3114 |
| pH | 0.3218 | **0.0011** | 0.1732 | **0.0091** | 0.6186 | **0.0001** | 0.0309 | 0.3465 | 0.5338 | 0.6547 |
| Soil conductivity | 0.0610 | 0.2340 | 0.4017 | **0.0001** | -0.0454 | 0.3877 | 0.3048 | **0.0031** | 0.4846 | 0.6497 |
| Total phosphorus | 0.3126 | **0.0005** | 0.0793 | 0.0972 | 0.6549 | **0.0001** | -0.0306 | 0.4139 | 0.5346 | 0.6801 |
| Total nitrogen | -0.0102 | 0.4937 | 0.2670 | **0.0050** | 0.4204 | **0.0001** | 0.0691 | 0.1838 | 0.4629 | 0.5660 |
| NO_3_-nitrogen | 0.3199 | **0.0003** | 0.1017 | **0.0500** | -0.0503 | 0.3269 | 0.2439 | **0.0040** | 0.4593 | 0.5744 |
| NH_4_-nitrogen | 0.1084 | 0.0993 | 0.3992 | **0.0001** | -0.0055 | 0.5814 | 0.2901 | **0.0044** | 0.5019 | 0.6457 |
| organic matter | 0.1852 | **0.0242** | -0.0874 | 0.1545 | 0.5196 | **0.0002** | 0.1182 | 0.0903 | 0.4842 | 0.6007 |
| Cl^-^ | 0.3256 | **0.0011** | 0.1466 | **0.0200** | 0.4521 | **0.0001** | 0.0605 | 0.2098 | 0.4794 | 0.6275 |
| SO_4_^2-^ | 0.0944 | 0.1175 | -0.0275 | 0.3889 | 0.6275 | **0.0001** | -0.0097 | 0.4944 | 0.4808 | 0.6253 |
| Na^+^ | -0.0304 | 0.4429 | 0.3828 | **0.0003** | -0.0517 | 0.3110 | 0.2561 | **0.0016** | 0.4720 | 0.6357 |
| K^+^ | 0.1819 | **0.0267** | -0.0903 | 0.1534 | 0.2998 | **0.0036** | 0.0960 | 0.1092 | 0.4752 | 0.6312 |
| Mg^2+^ | 0.0598 | 0.2044 | 0.0887 | 0.1373 | 0.2817 | **0.0090** | 0.0994 | 0.0985 | 0.4516 | 0.5814 |
| Ca^2+^ | 0.0382 | 0.3015 | 0.1509 | **0.0336** | 0.6612 | **0.0001** | -0.0369 | 0.3920 | 0.4714 | 0.6145 |

Note: *P* values less than 0.05 are bolded. Euclidean genetic distance matrices and difference matrices describing the differences in soil parameters among individuals were used.


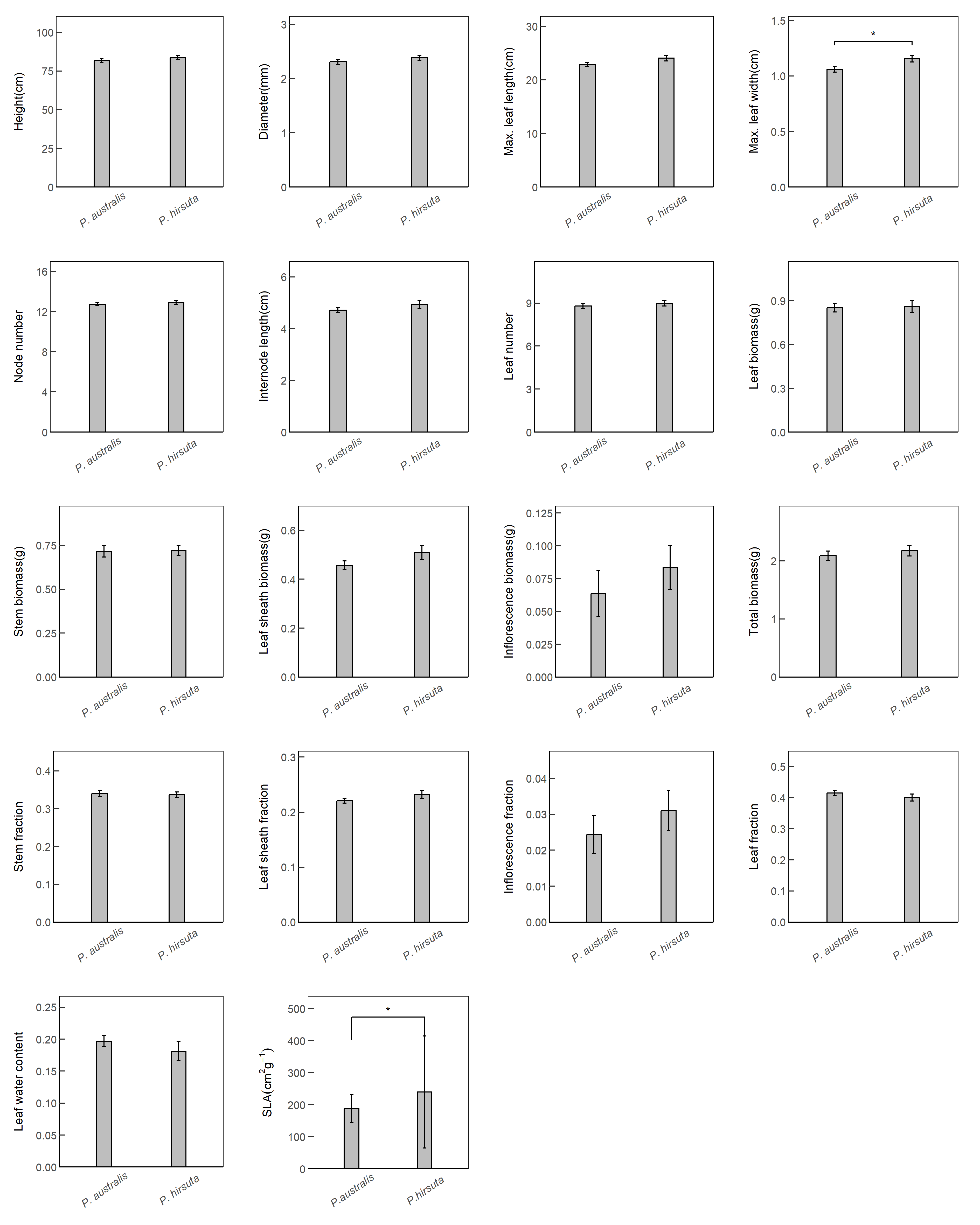


A**.**


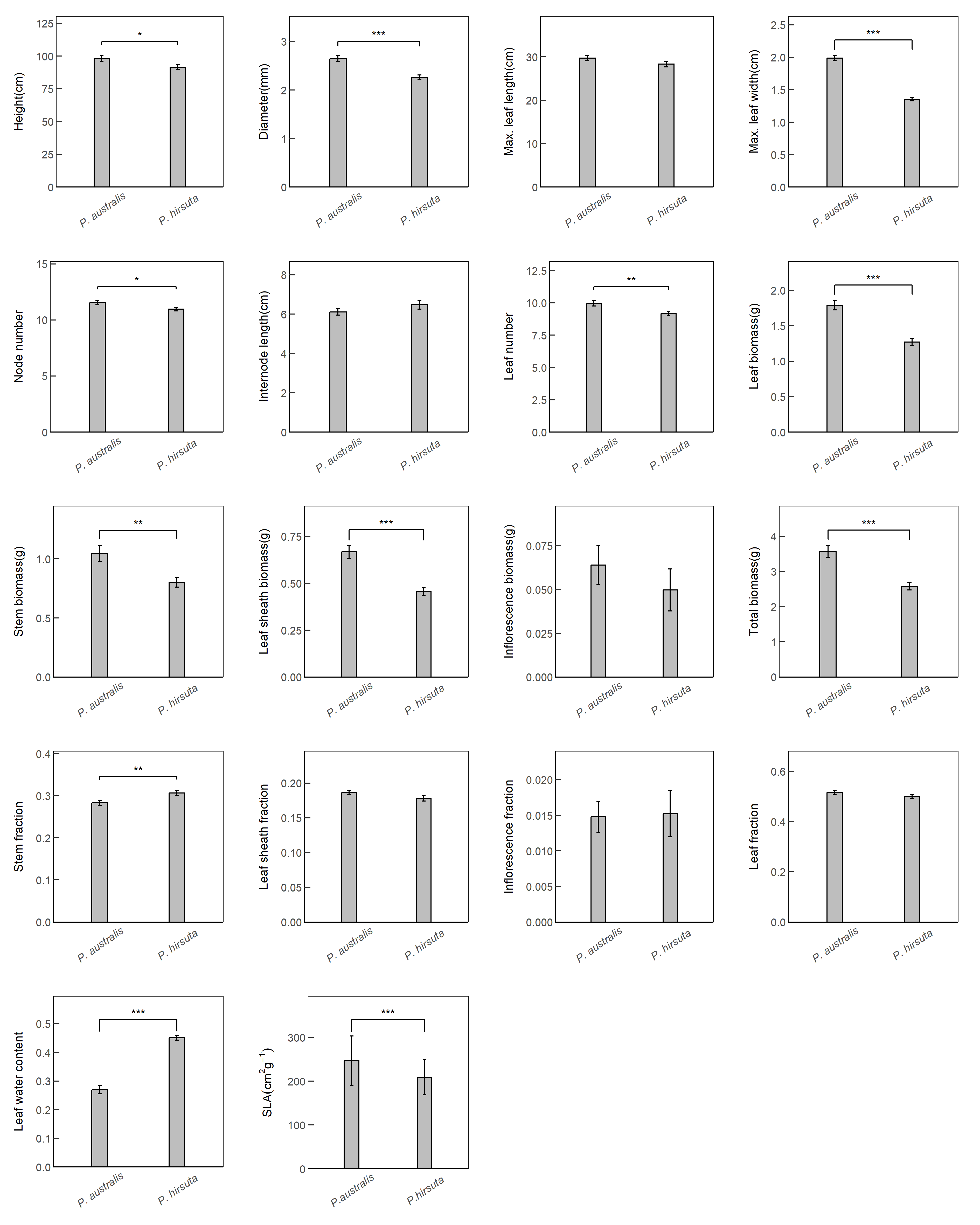
B.

**Figure S1** Comparison between *P. australis* and *P. hirsuta* in saline-alkaline meadow soil (SAS) (A) and sandy soil (SS) (B).

Note: Asterisks indicate the significance of pairwise comparisons (Student’s *t* test; *P<0.05; **P < 0.01).


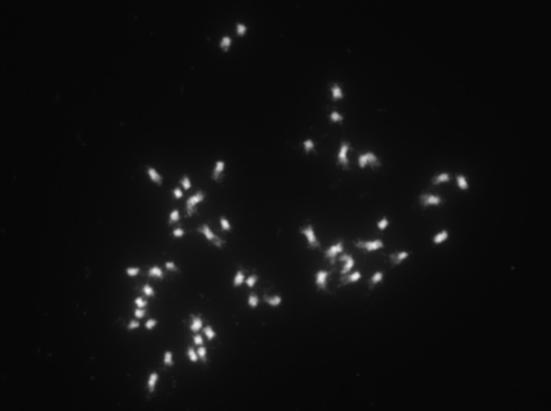

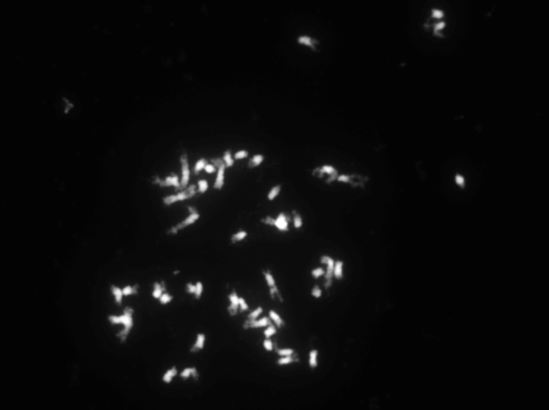


A. B.

**Figure S2** Somatic metaphase chromosomes of *Phragmites australis* (A) and *Phragmites hirsuta* (B) (2n=4x=48)


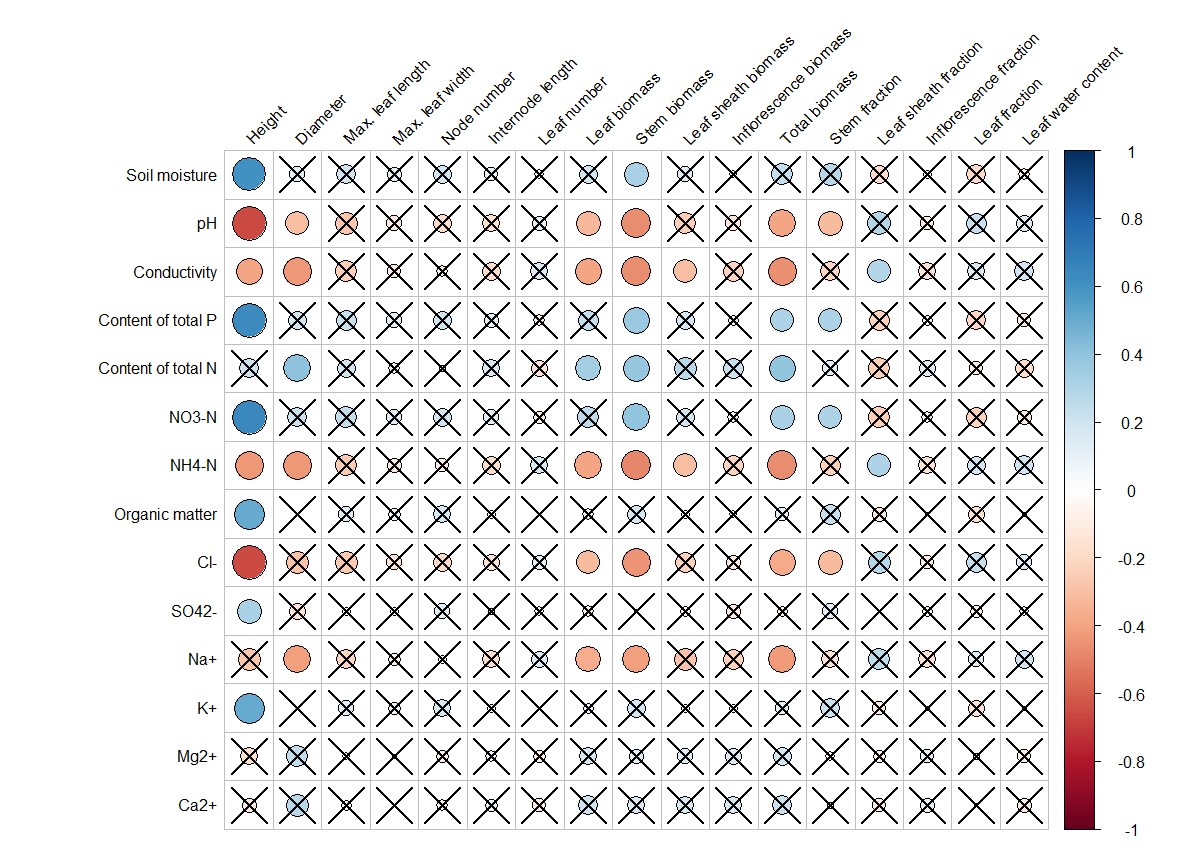
**
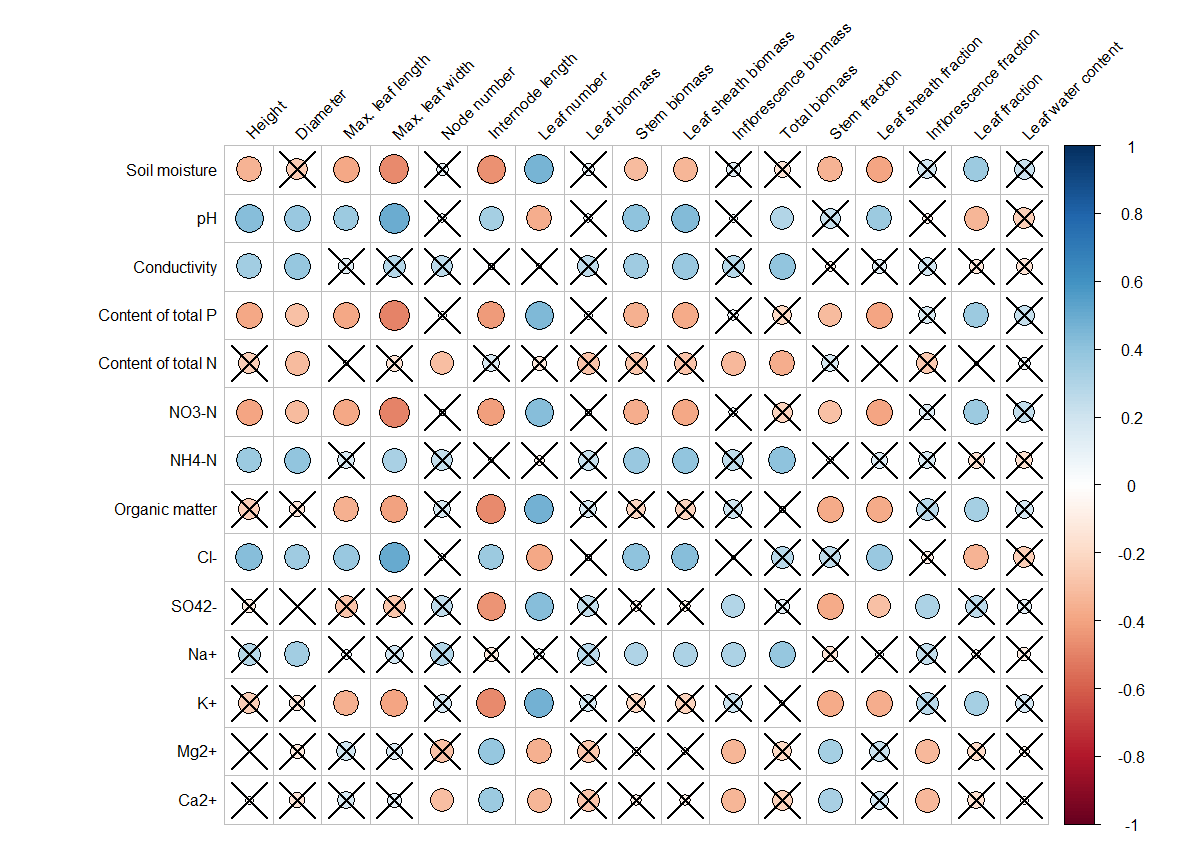
**


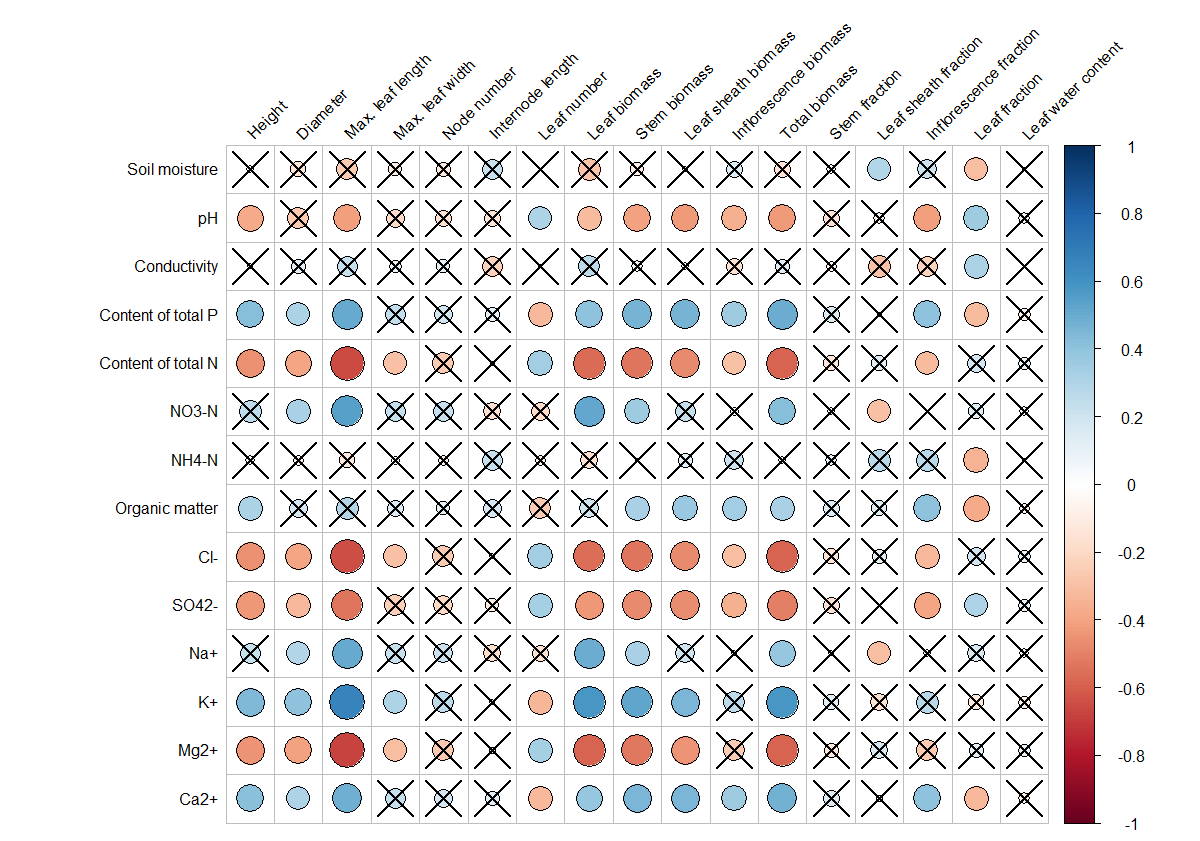
A. B.


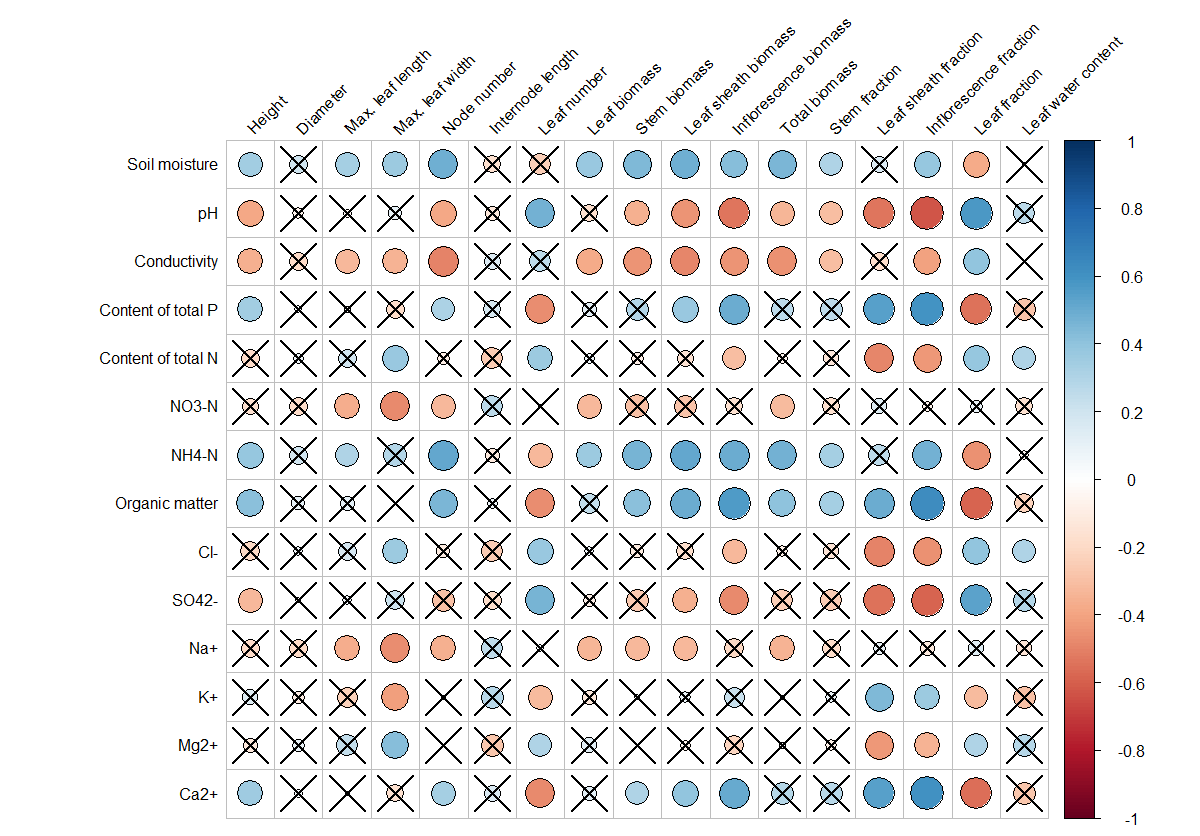


C. D.

**Figure S3** Correlations between phenotypic traits and soil characteristics of *P. australis* (A) and *P. hirsuta* (B) in saline-alkaline meadow soil (SAS) and *P. australis* (C) and *P. hirsuta* (D) in sandy soil (SS). Correlations were either positive (blue) or negative (orange) with circle symbols, varied in strength (size of shapes according to correlation coefficients) and had different levels of support (denoted as nonsignificant with a cross or significant (P<0.05) without a cross).
